# Microstructure-Based Modeling of Deformation and Damage Behavior of Extruded and Additively Manufactured 316L Stainless Steels

**DOI:** 10.3390/ma17102360

**Published:** 2024-05-15

**Authors:** Huai Wang, Ho-Won Lee, Minh Tien Tran, Dong-Kyu Kim

**Affiliations:** 1 School of Mechanical and Automotive Engineering, Qingdao University of Technology, Qingdao 266520, China; 2 Department of Materials AI & Big Data, Korea Institute of Materials Science, Changwon 51508, Republic of Korea; h.lee@kims.re.kr (H.-W.L.); mttran@kims.re.kr (M.T.T.); 3 Department of Mechanical & Aerospace Engineering, Konkuk University, Seoul 05029, Republic of Korea

**Keywords:** additive manufacturing, crystal plasticity, microstructure, mechanical behavior, damage, stainless steel

## Abstract

In this study, we investigated the micromechanical deformation and damage behavior of commercially extruded and additively manufactured 316L stainless steels (AMed SS316L) by combining experimental examinations and crystal plasticity modeling. The AMed alloy was fabricated using the laser powder bed fusion (LPBF) technique with an orthogonal scanning strategy to control the directionality of the as-fabricated material. Optical microscopy and electron backscatter diffraction measurements revealed distinct grain morphologies and crystallographic textures in the two alloys. Uniaxial tensile test results suggested that the LPBFed alloy exhibited an increased yield strength, reduced elongation, and comparable ultimate tensile strength in comparison to those of the extruded alloy. A microstructure-based crystal plasticity model was developed to simulate the micromechanical deformation behavior of the alloys using representative volume elements based on realistic microstructures. A ductile fracture criterion based on the microscopically dissipated plastic energy on a slip system was adopted to predict the microscopic damage accumulation of the alloys during plastic deformation. The developed model could accurately predict the stress–strain behavior and evolution of the crystallographic textures in both the alloys. We reveal that the increased yield strength in the LPBFed alloy, compared to that in the extruded alloy, is attributed to the higher as-manufactured dislocation density and the cellular subgrain structure, resulting in a reduced elongation. The presence of annealing twins and favorable texture in the extruded alloy contributed to its excellent elongation, along with a higher hardening rate owing to twin–dislocation interactions during plastic deformation. Moreover, the grain morphology and defect state (e.g., dislocations and twins) in the initial state can significantly affect strain localization and damage accumulation in alloys.

## 1. Introduction

Additive manufacturing (AM) has emerged as a revolutionary manufacturing technology with the potential to transform various industries. Its application to metallic materials is particularly noteworthy, which offers unparalleled advantages such as design flexibility, lowered material waste, and the ability to produce complex geometries with improved performance characteristics [1,2,3]. AM technologies suitable for processing metallic materials can be broadly classified into two types: powder bed fusion (PBF) and direct energy deposition (DED). PBF employs laser or electron beam as the heat source to selectively melt metal particles and build layer-by-layer in a powder bed, whereas DED uses a focused energy source to deposit material in the form of a powder or wire onto a substrate or previously deposited layer. Through the optimization of processing conditions, these AM techniques were shown to be able to tailor the microstructures and achieve superior mechanical and/or anti-corrosion properties, compared to those produced by conventional casting or wrought processes, in various metallic material systems, including steels [4], aluminum alloys [5], titanium alloys [6], and magnesium alloys [7].

Austenitic 316L stainless steel (SS316L) has long been a ‘workhorse material’ with wide-ranging engineering applications owing to its excellent corrosion and oxidation resistances. Through a precise control of processing parameters, such as the laser power, hatch spacing, scanning speed, and powder characteristics—which are critical parameters in determining the microstructure, mechanical properties, and structural integrity—AM can be used to develop designated microstructures to achieve exceptional mechanical and anti-corrosion properties in SS316L [4,8,9]. For instance, AMed SS316L processed via laser-based PBF exhibited an ultimate tensile strength (UTS) of 640 MPa and a uniform elongation of 59% [4]. It was evidenced that the high strength of the LPBFed alloy was mainly achieved by its solidification-enabled subgrain cellular structures instead of HAGBs, in which the cellular wall thickness was considered critical. Moreover, the highly uniform elongation was correlated with the steady and progressive work-hardening behavior regulated by its hierarchically heterogeneous microstructure. In another study, the nanotwinning mechanism, promoted by favorable crystallographic textures, was found to be able to improve both the strength and ductility of LPBF-built SS316L; this has been attributed to the low twin-boundary energy and the fact that dislocations are able to pass through or glide along the twin boundaries by dissolving into partial dislocations [10]. Owing to the distinct thermal history and complex solidification kinetics involved in AM processes, additively manufactured SS316L typically exhibits a layered mesoscale morphology and hierarchical microstructure comprising fine columnar grains, cellular dislocation tangles, and nanoinclusions [3,10,11]. To date, extensive studies have examined meso- and microscale characteristics (such as the defect state, phase, texture, and grain/interface morphologies) and established their relationships with the mechanical (such as the yield/tensile strength, fatigue, and creep), corrosive, and other properties of AMed SS316L [12,13,14,15,16,17,18].

The AM process induces hierarchical heterogeneities, resulting in complex micromechanical behaviors during plastic deformation. Therefore, phenomenological constitutive models, which can accurately predict the macroscopic deformation behavior of conventionally processed metallic materials, struggle to capture the profound anisotropic mechanical responses of AMed SS316L when subjected to external loading. Micromechanical modeling based on the crystal plasticity theory has emerged as a versatile alternative. This approach quantitatively links the macroscopic deformation behavior of crystalline materials to the microscopic deformation mechanism by taking into account microstructural features such as grain morphology, crystallographic texture, and phase/grain boundaries [19,20]. Furthermore, microscopic fracture models related to various failure mechanisms (interface delamination, matrix fracture, and inclusion fracture), including cohesive models, plasticity energy dissipation, and accumulated plastic shear and fatigue initiation parameters, have been proposed and recently incorporated into crystal plasticity frameworks to predict the microscopic strain localization and damage the accumulation behavior governed by microstructural features in metallic materials [21,22,23,24,25,26]. Studies have demonstrated that the crystallographic texture and grain morphology are two key factors in AMed SS316L that influence its macroscopic anisotropy, deformation incompatibilities, stress, and slip transfer at grain boundaries [27,28,29]. For a complete understanding of the strain localization and damage accumulation behavior of this material, it is essential to perform crystal plasticity modeling using a realistic microstructure of AMed SS316L. However, most studies have utilized synthetic representative volume elements (RVEs) to simulate the deformation behavior of AMed SS316L [28,29,30], with the mechanisms underlying the strain localization and damage accumulation remaining unclear.

In this study, we integrated the crystal plasticity theory with an energy-based micromechanical damage criterion for a comparative analysis of the micromechanical deformation and damage behavior of commercially extruded steel and AMed SS316L (using the LPBF technique). We developed a mapping algorithm to generate RVEs based on the realistic microstructures of the alloys; the RVEs were subsequently employed to accurately replicate the deformation behavior of the studied materials. Through a combination of experimental and numerical analyses, we established microstructure–property linkages for the two alloys and elucidated the dependence of the deformation behavior, strain localization, and damage accumulation on the microscopic characteristics.

## 2. Experimental Procedure

### 2.1. Material Preparation

**Figure 1:**
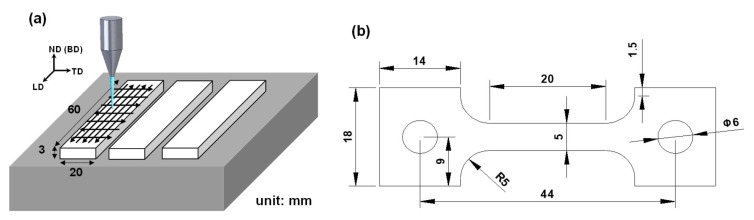
Illustration of (**a**) scanning strategy and geometry of LPBF blocks; (**b**) dimensions of the LPBF tensile specimens.

AMed SS316L was fabricated using the LPBF technique by employing a Concept Laser M2 machine (Concept Laser, Lichtenfels, Germany) under an inert argon gas atmosphere at a pressure of 10 mbar and an oxygen content of 0.2%. The as-received gas-atomized alloy powder has a particle size ranging from 45 to 150 µm. Table 1 presents its nominal chemical composition. During the LPBF process, the laser power, scanning speed, layer thickness, and hatch width (laser beam spot size) were, respectively, 250 W, 1.5 m/s, 250 µm, and 400 µm. As shown in Figure 1a, three blocks measuring 20 mm (TD) × 3 mm (ND) × 60 mm (LD) were constructed by using an orthogonal scanning strategy. Subsequently, the as-built blocks were cut into tensile specimens using electric discharge machining; the dimensions provided in Figure 1b are for the LPBFed alloy (LA). For the extruded samples, dog bone-shaped tensile specimens with a gauge length of 50 mm and radius of 12.5 mm were prepared from commercially annealed extruded alloy (EA) bars in accordance with ASTM E8/E8M standards [31].

### 2.2. Tensile Test and Microstructural Examination

Uniaxial tensile tests were conducted on the prepared specimens with an initial engineering strain rate of 0.001 s^−1^ to characterize their tensile properties, including the yield strength (YS), UTS, elongation to fracture, and strain-hardening behavior, of both the LA and EA. The normal direction (ND) was parallel to the building direction (BD), and the loading axis was aligned with the longitudinal direction (LD) of the LPBF specimen. Throughout each test, the deformation of the specimens was monitored using a digital image correlation (DIC) technique facilitated by an ARAMIS 3D 5M system (GOM Metrology, Braunschweig, Germany). The tensile tests were repeated thrice for each alloy to ensure repeatability of the results.

Microstructural observations of both the alloys were conducted on planes perpendicular to the LD and ND using optical microscopy (OM), and electron backscatter diffraction (EBSD) measurements were performed inside a field-emission scanning electron microscope (JEOL-7100F, Tokyo, Japan). The samples were mounted in resin, ground with SiC paper, and polished electromechanically. The microstructures and crystallographic textures of the initial and fractured states were examined. To analyze the microstructures, small cubic samples were extracted from both the undeformed and deformed regions of the tensile specimens in the mid-thickness layer. The EBSD analysis was outperformed using a step size of 3.0 μm for the EA and 2.0 μm for the LA. The obtained EBSD data for the tested alloys were analyzed using EDAX TSL OIM Analysis (ver. 7.2).

## 3. Numerical Modeling

### 3.1. Crystal Plasticity Framework

The rate-dependent crystal plasticity constitutive model developed in this study follows the framework originally proposed by Rice et al. [32,33], which is briefly described below. The total deformation of a crystalline material is assumed to be caused by dislocation slips on active slip systems and the elastic distortion of the crystal lattice. The elastic constitutive equation for a single crystal is as follows:

(1) $$\sigma^{\nabla }=C∶D-{\dot{\sigma }}^{0}-\sigma tr\left(D\right),$$

where $\sigma^{\nabla }$ is the Jaumann rate of the Cauchy stress, D represents the strain rate tensor, C is the fourth-order tensor of the elastic modulus, and ${\dot{\sigma }}^{0}$ is the viscoplastic-type stress rate, which is determined by the slip rates on the various slip systems in the crystal.

The slip rate on the $\alpha^{th}$ slip system is assumed to obey Schmid’s law and is driven by the resolved shear stress $\tau^{\alpha }$:

(2) $${\dot{\gamma }}^{\alpha }={\dot{\gamma }}_{0}^{\alpha }{\left|\tau^{\alpha }/\tau_{c}^{\alpha }\right|}^{1/m}\mathrm{sgn}\left(\tau^{\alpha }\right),$$

where ${\dot{\gamma }}_{0}^{\alpha }$ is the reference shear rate assumed to be the same for all slip systems, $\tau_{c}^{\alpha }$ represents the critical resolved shear stress (CRSS) for the system, and m is the coefficient of strain rate sensitivity.

The strain hardening of the slip system can be characterized by the evolution of the CRSS and is determined by

(3) $${\dot{\tau }}_{c}^{\alpha }=\sum_{\beta }h^{\alpha \beta }\left|{\dot{\gamma }}^{\beta }\right|,$$

where $h^{\alpha \beta }$ is the hardening matrix, which considers the interaction between the slip systems including self-hardening (α=β) and latent-hardening (α≠β). It can be expressed as follows:

(4) $$h^{\alpha \beta }=qh^{\alpha \alpha }+\left(1-q\right)h^{\alpha \alpha }\delta_{\alpha \beta },$$

where $h^{\alpha \alpha }$ is the self-hardening modulus, $\delta_{\alpha \beta }$ is the Kronecker delta, and q is the latent-hardening rate due to the impact of slip activity on other slip systems. The hardening law for a slip system is defined as follows [34]:

(5) $$h^{\alpha \alpha }=h_{0}{\left(1-\frac{\tau_{c}^{\alpha }}{\tau_{s}}\right)}^{a}.$$

To model the micromechanical ductile fracture behavior, we followed the suggestion of refs. [26,35], which outlined that the initiation of a fracture in polycrystals is governed by the plastic energy dissipated on a critical slip plane in the framework of crystal plasticity modeling. Therefore, a microscopic ductile fracture criterion in terms of the dissipated plastic energy at the grain scale was adopted and implemented in the aforementioned crystal plasticity framework to simulate the initiation of ductile fracture caused by the accumulation of strain localization. The criterion for microscopic plastic energy dissipation is related to the plastic energy dissipated over all the slip systems due to dislocation slips [23,35]:

(6) $$G_{p}=\int_{0}^{t}\sum_{\alpha =1}^{N}\left|\tau_{c}^{\alpha }{\dot{\gamma }}^{\alpha }\right|dt,$$

where ${\dot{\gamma }}^{\alpha }$ and $\tau_{c}^{\alpha }$ are, respectively, the shear strain rate and CRSS for a slip system, and *N* is the total number of slip systems. Once $G_{p}$ reaches a critical value, damage will be initiated in the model. To study micromechanical deformation, the accumulated slip (Γ) and stress triaxiality (η) defined in Equations (7) and (8) are also used as indicators for the strain localization and damage behavior of the material, as the accumulated slip is closely related to the strain localization behavior [21] and since higher stress triaxialities are known to facilitate the growth of voids during ductile fracture processes [36].

(7) $$\Gamma =\int_{0}^{t}\sum_{\alpha =1}^{N}\left|{\dot{\gamma }}^{\alpha }\right|dt,$$

(8) $$\eta =\frac{\frac{1}{3}\left(\sigma_{1}+\sigma_{2}+\sigma_{3}\right)}{{\bar{\sigma }}_{eq}},$$

where $\sigma_{1}$, $\sigma_{2}$, and $\sigma_{3}$ denote the three principal stresses, respectively, and ${\bar{\sigma }}_{eq}$ is the von Mises equivalent stress.

### 3.2. Microstructure-Based Finite Element Model

**Figure 2:**
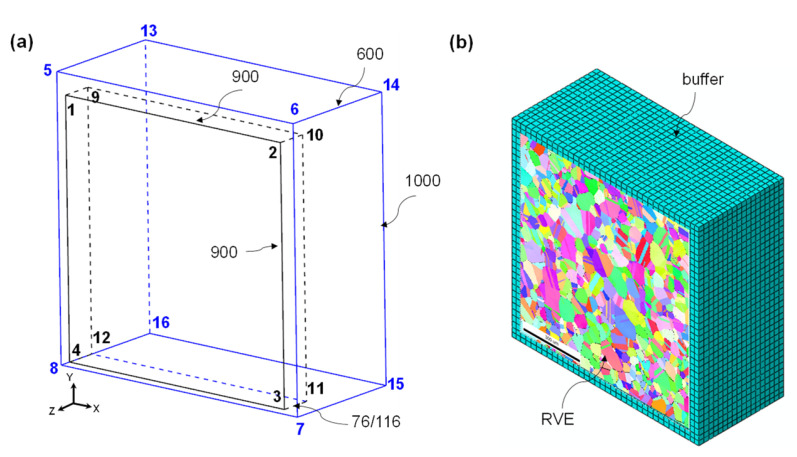
(**a**) Geometry (blue line: outline of the buffer region, black line: outline of the CPFE region); (**b**) FE mesh of a microstructure-based representative volume element (RVE) model.

The previously described crystal plasticity constitutive and ductile fracture models were implemented in the user material subroutine (UMAT) for use in finite element packages, such as Abaqus/Standard 6.14. These models were employed to simulate the micromechanical deformation behavior of the two alloys. Crystal plasticity finite element (CPFE) simulations were conducted using microstructure-based RVEs, which directly mapped the microstructure obtained from the EBSD (see Figure 2), to examine the micromechanical plastic deformation, strain localization, and microscopic damage behavior subjected to uniaxial tension. To generate the microstructure-based mapped RVEs, the crystallographic texture of a material, represented by Euler angles (*φ*_1_, *Ф*, and *φ*_2_), and their corresponding coordinate information obtained from EBSD experiments were utilized as the input. The SDVINI subroutine was integrated into the UMAT subroutine to assign crystallographic texture information to an FE mesh in the RVE mapping region. Within the developed subroutine, the discrepancy between the coordinates from the EBSD data points and those of the FE mesh was calculated to find the nearest material point and assign the corresponding Euler angles to integration points.

The center part of the detected EBSD region, as highlighted by the black dash-lined rectangle in the inverse pole figure (IPF) maps in Figure 5c,d, with a dimension of 900 μm × 900 μm, was chosen to represent the initial microstructure of the alloys. This selected area was used to map the grain information onto an FE mesh. More importantly, the microstructure-based mapped RVE was encased in a buffer region. By using this method, the FE model ensures more natural and realistic boundary conditions during the micromechanical simulations [23]. The dimensions of the buffer regions were 1000 μm × 1000 μm × 600 μm. The thickness of the mapped RVE was determined such that there was only one grain throughout the thickness of the RVE, maintaining a thickness-to-grain-size ratio (*t*/*d*) of 1. The thickness was set to be 76 μm for the EA and 116 μm for the LA according to the EBSD results presented in Section 4.1.

**Figure 3:**
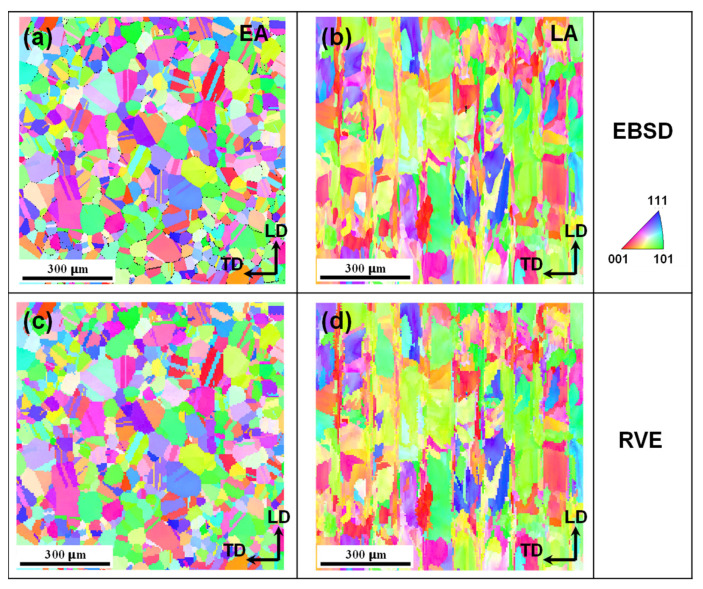
Inverse pole figure maps of the two alloys studied: (**a**,**b**) selected regions from EBSD data; (**c**,**d**) relevant mapped RVEs.

For the computational cost, the isotropic von Mises plasticity criterion and the Swift-hardening law were assigned to the buffer region, whereas the mapped RVE region was simulated using the rate-dependent crystal plasticity constitutive law described above using the UMAT subroutine. The eight-node solid element with a reduced integration (C3D8R) was adopted to generate the FE mesh, and the total numbers of elements for the buffer and RVE regions were, respectively, 68,400 and 253,125 for the EA and 64,350 and 253,125 for the LA. Figure 3 shows the IPFs of the mapped RVE for the two alloys, indicating the effectiveness of the mapping method in reproducing the real microstructures of the alloys. To simulate the uniaxial tensile tests, periodic boundary conditions (PBCs) were applied to the four planes (5-8-16-13, 5-6-14-13, 6-7-15-14, and 7-8-16-15) of the buffer region, while tie constraints were applied to the five planes (1-4-12-9, 3-4-12-11, 2-3-11-10, 1-2-10-9, and 9-10-11-12) of the alloys. A symmetry boundary condition was applied to the back plane, as indicated by the (13-14-15-16) plane of the buffer region, whereas both the front planes, as indicated by the (1-2-3-4) and (5-6-7-8) planes of the buffer and RVE regions, respectively, were free from out-of-plane normal and shear constraints [23].

## 4. Results and Discussion

### 4.1. Initial Microstructure and Crystallographic Texture

**Figure 4:**
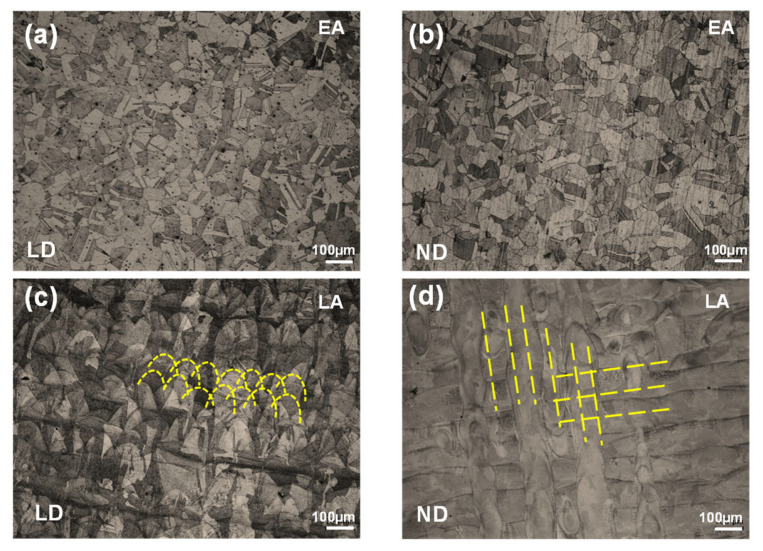
Optical microscopy (OM) images of the initial microstructures examined on (**a**) the LD plane of EA, (**b**) the ND plane of EA, (**c**) the LD plane of LA, and (**d**) the ND plane of LA.

**Figure 5:**
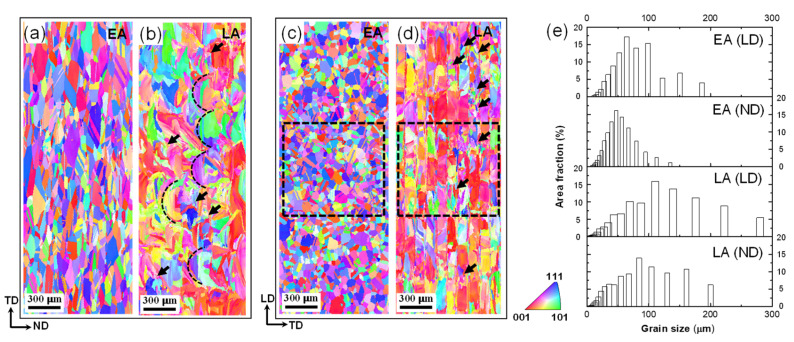
Inverse pole figure maps obtained by electron backscatter diffraction (EBSD) on (**a**) the LD plane of EA, (**b**) the LD plane of LA, (**c**) the ND plane of EA, (**d**) the ND plane of LA, and (**e**) grain size distributions for the two alloys. Note: the dotted boxes in (**c**,**d**) represent the selected region for mapping the Euler angles used in CPFE analysis.

Figure 4 and Figure 5 show the initial microstructures of the EA and LA observed in the LD and ND planes. The EA exhibits an equiaxed grain morphology with some visible annealing twins, with an average grain size of approximately 76.6 μm on the ND plane and 50.9 μm on the LD plane, respectively. In contrast, the LA exhibits a partially elliptic morphology on the LD plane due to the melt pool, as indicated by the dashed elliptic curves in Figure 4c and Figure 5b, in addition to an orthogonal path-like morphology on the ND plane resulting from the scanning path of the LPBF process, as indicated by the dashed lines in Figure 4d and the clear interfaces between the paths in Figure 5d. Moreover, substructures (e.g., small grains) can be observed inside the melt pools in the LA, which are highlighted by dark arrows in Figure 5b,d; these can be attributed to the varying thermal gradients experienced by different regions. Owing to the LPBF-induced hierarchical microstructure, the average grain size of the LA measures approximately 116.4 μm on the ND plane and 84.9 μm on the LD plane, with relative uncertainties (defined as the ratio of the standard error to the absolute average value) of 28.5% and 60.3%, respectively. The high uncertainty means that the sizes of the constituent grains in the LA are quite dispersed (see also in Figure 5e), which is a result of the hierarchical nature of the microstructure of the LA.

**Figure 6:**
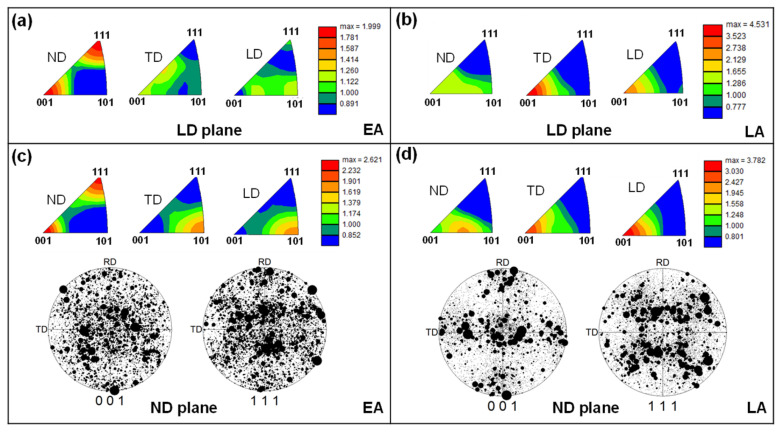
Inverse pole figures and pole figures of the two alloys: (**a**,**c**) EA; (**b**,**d**) LA.

Figure 6 shows the IPFs and pole figures measured in the LD and ND planes for the two alloys. The pole figures were plotted using a single point for each grain weighted by size. The analysis revealed that EA exhibits a rotated cube and Goss texture, with strong <100> and <111> textures along the ND and weaker <110> textures along the TD and LD. In contrast, the LA exhibited a strong <100> texture along the TD and LD and a weaker <102> texture along the TD. Notably, the <110> texture along the LD of the EA is favorable for twinning, whereas the strong <100> texture along the LD of the LA is unfavorable for twinning [10].

**Figure 7:**
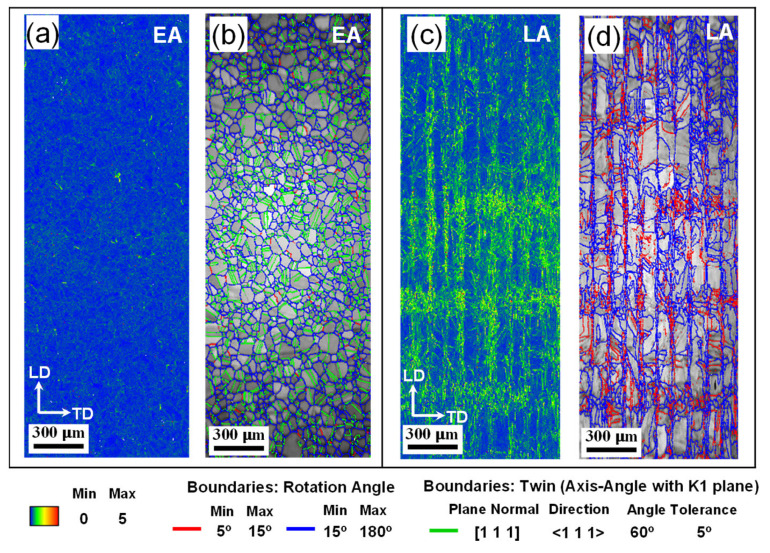
Initial KAM and grain boundary maps of the two alloys measured on the ND plane of (**a**,**b**) EA and (**c**,**d**) LA.

Figure 7 shows the Kernel average misorientation (KAM) and grain boundary maps of the two alloys on the LD plane. The EA showed a lower initial KAM value than the LA. Higher KAM values were concentrated around the interface between the scanning paths and the melt pools. The fractions of low-angle and high-angle grain boundaries (LAGBs and HAGBs) were 3.6% and 96.4%, respectively, for the EA, whereas these fractions were 48.6% and 51.4% for the LA, respectively. Notably, the EA exhibited a significant number of annealing twins, as indicated by Figure 7b, with a fraction of 44.2%. The disparity in the initial defect states contributes to the varied mechanical properties of the two alloys [4,10,13], which is further discussed in Section 4.2, Section 4.3 and Section 4.4.

### 4.2. Stress–Strain Behavior and Deformed Microstructure

**Figure 8:**
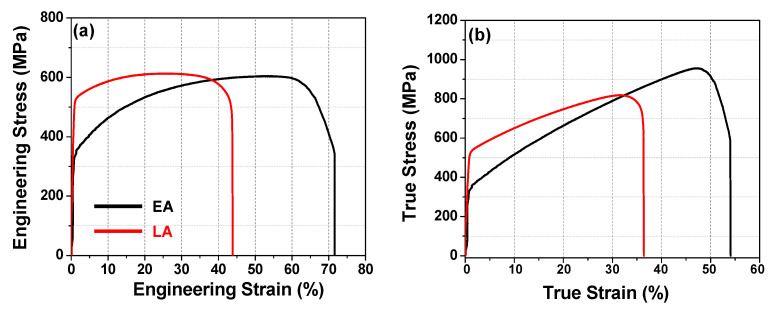
Engineering (**a**) and true (**b**) stress–strain curves of the two alloys.

Figure 8a,b show the engineering and true stress–strain curves of the two alloys, respectively. Evidently, the LA exhibits a significantly higher yield strength (516 MPa) than the EA (328 MPa). Conversely, the EA demonstrates a higher elongation (71%) than the LA (44%). The UTS of the EA (604 MPa) was slightly lower than that of the LA (612 MPa), as summarized in Table 2. The higher strength of the LA can be attributed to its higher dislocation density and the solidification-enabled subgrain cellular structures, as indicated by the KAM map in Figure 7c,d, which is consistent with previous findings [4,11,37]. In Table 2, the mechanical properties of the LPBEed SS316L alloy obtained in several previous studies are also presented. Similar conclusions have been drawn in [4,11] with regard to the simultaneously enhanced strength and ductility of the LPBEed SS316L alloys being due to the concurrent contribution by dislocation slips, cellular substructures, and deformation twinning. The lower YS and the UTS of the developed LA achieved in the present study and that in [38] is probably due to the absence of deformation twinning, which was inhibited by the development of an unfavored texture [10]. In contrast, the superior elongation and the high work hardening of the EA is attributed to its favorable crystallographic texture for twinning, which can concurrently enhance both the strength and ductility of the material [10].

**Figure 9:**
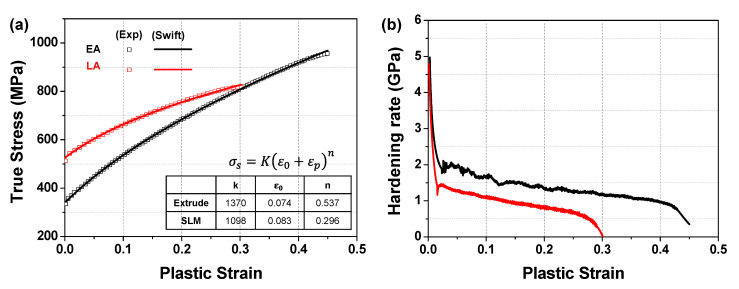
Strain-hardening behavior of the two studied alloys: (**a**) yield stress; (**b**) hardening rate as a function of the plastic strain.

**Figure 10:**
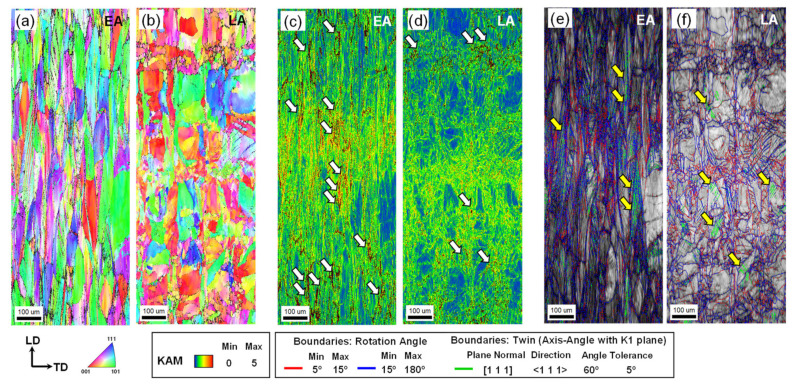
Deformed microstructures of the two studied alloys measured on the ND plane: (**a**,**b**) IPF maps; (**c**,**d**) KAM maps; (**e**,**f**) grain boundary maps. (Note: white and yellow arrows indicate the concentration of dislocations and twins, respectively).

Figure 9 illustrates the strain-hardening behaviors of the two alloys. Evidently, the EA exhibited a higher hardening rate than the LA. This behavior is attributed to the presence of annealing twins in a significant portion of the grains in the EA, leading to numerous twin–dislocation interactions during plastic deformation and better elongation [4,10], as depicted in Figure 10a and indicated by yellow arrows in Figure 10e. As shown in Figure 10c, the strain localization behavior of the EA is relatively uniform, with strain localization occurring simultaneously at multiple sites. Conversely, in the case of the LA, the presence of more as-manufactured dislocations resulted in a higher yield strength but significantly reduced elongation due to severe premature strain and damage localization at multiple sites, as shown in Figure 10c. After tensile fracture, as illustrated in Figure 10e,f, the fraction of LAGBs increases from 51.4% to 71% in the LA, whereas in the EA, it increases from 3.6% to 63.5%. This is indicative of a notable textural change in the EA, which is further discussed in Section 4.3.

### 4.3. Microstructure-Based Modeling of the Tensile Deformation Behavior

**Figure 11:**
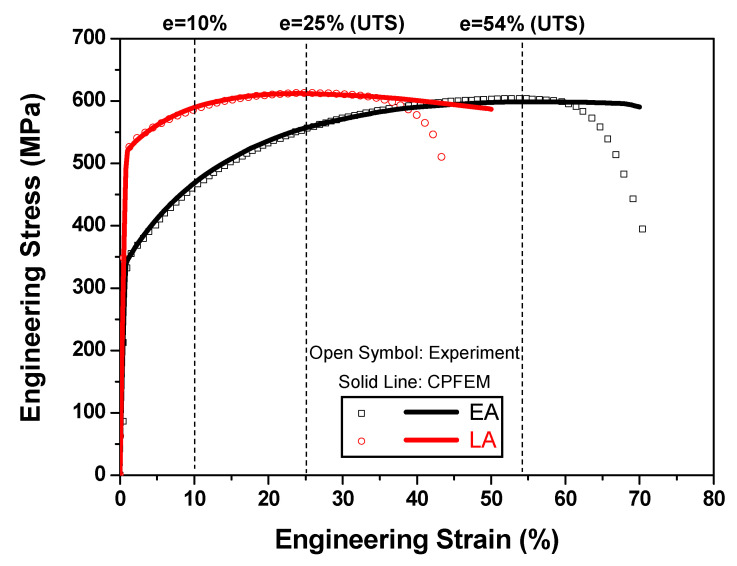
Engineering stress–strain curves of the two studied alloys predicted by the crystal plasticity finite element (CPFE) model.

The developed CPFE model and microstructure-based RVEs described in Section 3 were employed to simulate the tensile deformation and damage localization behaviors of the two alloys. In the CPFE simulations, the swift-hardening law was used for the buffer region to replicate the hardening responses of the alloys, as shown in Figure 9a. Subsequently, these responses were used to define the material parameters in FE simulations. Within the RVE region, the mechanical behavior was simulated using the crystal plasticity constitutive law, with the elastic constants for SS316L exhibiting an FCC crystal structure taken from [39] *C*_11_ = 204.6 GPa, *C*_12_ = 137.7 GPa, and *C*_44_ = 126.2 GPa. It was assumed that only the {111}<110> slip system was responsible for the plastic deformation of the alloys, and ${\dot{\gamma }}_{0}^{\alpha }$ and m were set to 0.001 s^−1^ and 0.05, respectively. Tensile stress–strain curves were employed to calibrate the hardening parameters of the slip systems required to model the mechanical behavior of the alloys. Table 3 presents the calibrated material parameters. Figure 11 shows a comparison between the predicted and experimental stress–strain curves of the two alloys. The curves indicate that the developed microstructure-based CPFE model could accurately reproduce the uniaxial tensile deformation responses of the alloys.

**Figure 12:**
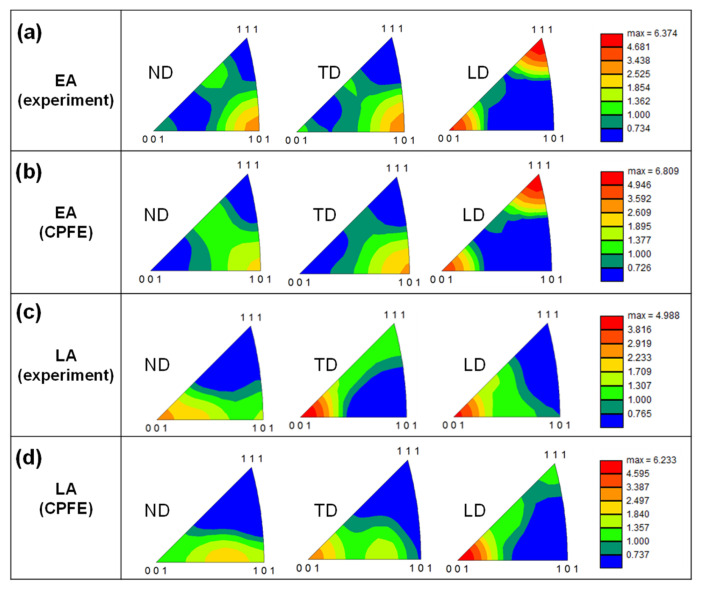
Inverse pole figures of the two alloys after fracture measured on the ND plane: (**a**) experimental results for EA, (**b**) CPFE predictions for EA, (**c**) experimental results for LA, and (**d**) CPFE predictions for LA.

Notably, the developed CPFE model helped predict the evolution of crystallographic textures. In Figure 12, we compare the IPFs of the materials at the moment of UTS (25% for LA and 54% for EA) with those measured by the EBSD after a tensile fracture. This comparison shows that the developed model could accurately predict the texture evolution during the tensile deformation of the two alloys. The texture of the EA underwent significant changes, with the <001> and <111> grains moving from the ND toward the LD (parallel to the loading direction), owing to the lowest Schmid factor along this direction. Conversely, the texture of the LA exhibited minimal changes because the early strain and damage location were insufficient to substantially modify the crystallographic orientation of the grains.

### 4.4. Effect of Microstructure on Strain Localization and Damage Behavior

**Figure 13:**
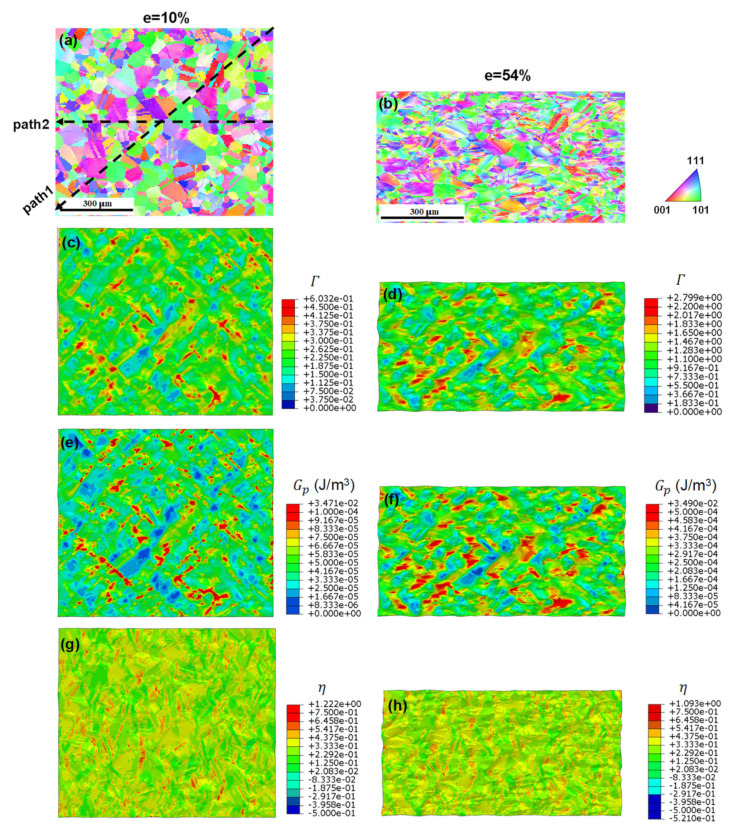
EA at two strain levels: (**a**,**b**) IPF maps; (**c**,**d**) distributions of the accumulated plastic slip; (**e**,**f**) plastic dissipation energy; (**g**,**h**) stress triaxiality.

To understand the microstructural influences on the deformation and damage behaviors of the alloys, Figure 13 illustrates the IPF maps and distributions of the accumulated plastic slip, microscopically dissipated plastic energy, and stress triaxiality on the front plane of the RVE model for the EA at two engineering strain levels: 10% and 54% (necking point). The IPF maps were generated by extracting the calculated current coordinates and Euler angles from the integration point of each element and manipulating a relevant “.ang” file. The evolution of the accumulated plastic slip serves as an indicator of strain localization; the dissipated plastic energy indicates micromechanical damage; the stress triaxiality reflects the stress concentration. The color distribution of the IPF was notably different from that of the initial state, as shown in Figure 5c. This is because the texture of the majority of the grains rotated toward the LD, as indicated in Figure 12a. At the selected strain levels (Figure 13g,h), the stress state appeared to be uniformly distributed across the examined region, and the stress concentration was not severe because of the equiaxed grain morphology of the EA. Consequently, the distribution of the strain localization sites appeared to be nearly random, even at an engineering strain of 54% (Figure 13d). Moreover, the distribution of the plastic energy dissipation generally aligned with the distribution of the accumulated plastic slip because the CRSSs in different individual grains for the {111}<110> slip system were assumed to be the same during the simulation. The homogeneous grain morphology and existence of prior twin boundaries are believed to contribute to the excellent tensile elongation of the alloy, and its tensile strength is comparable to that of LA [4,10,12,15].

**Figure 14:**
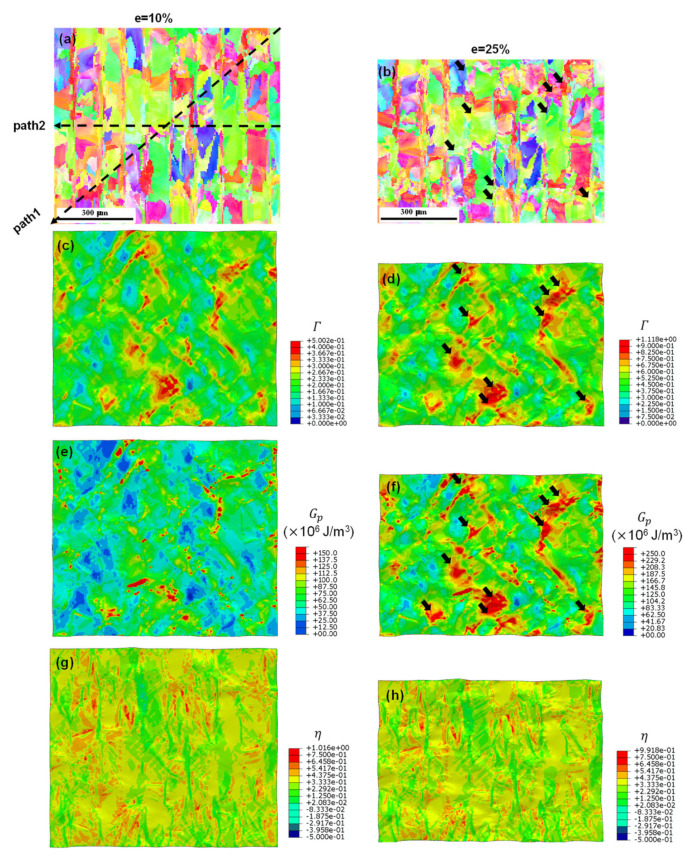
LA at two strain levels: (**a**,**b**) IPF maps; (**c**,**d**) distributions of the accumulated plastic slip; (**e**,**f**) plastic dissipation energy; (**g**,**h**) stress triaxiality.

Figure 14 shows the IPF maps and distributions of the accumulated plastic slip, microscopic plastic energy dissipation, and stress triaxiality on the front plane of the RVE model for the LA at two engineering strain levels: 10% and 25% (necking point). The IPF color scheme was similar to that of the initial state (Figure 3b), indicating that the majority of grains did not undergo significant changes in their orientation, which is consistent with the experimental findings presented in Figure 12c. Figure 14g,h reveal that the stress is predominantly concentrated at the interface between the different scanning paths owing to the distinctly different grain orientations, as indicated by the IPF color variation in Figure 14a. The accumulation of the plastic slip and dissipated plastic energy were also consistent, similar to the observations made for the EA. However, compared with the EA, the LA exhibited severe strain localization and damage accumulation in the different strain stages, as shown in Figure 14c–f. By comparing the KAM map (Figure 7c) and the grain boundary map (Figure 7d), we can identify two types of sites as locations for strain localization and damage accumulation in the LA: those with high initial dislocation densities and those with numerous HAGBs, as indicated by black arrows in Figure 14d,f.

**Figure 15:**
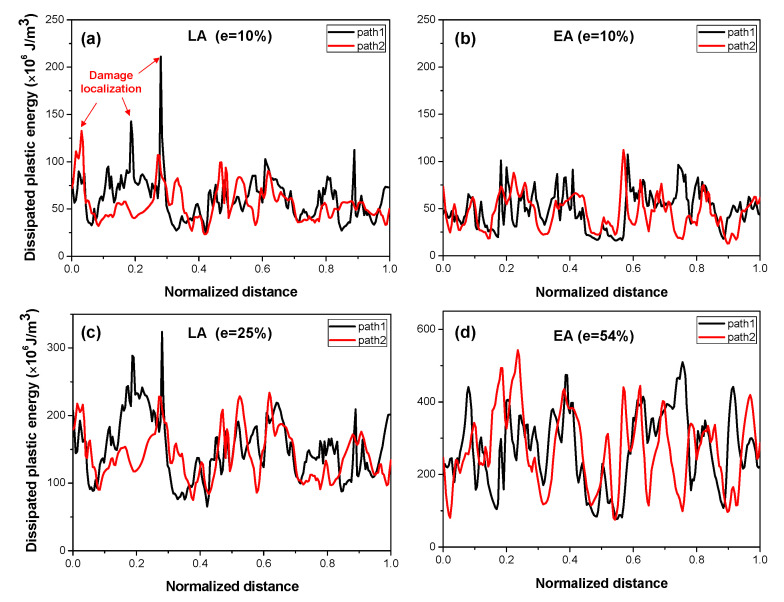
Distribution of the dissipated plastic energy along two paths in the RVE models of the two alloys at two strain levels: (**a**) LA (e = 10%), (**b**) EA (e = 10%), (**c**) LA (e = 25%), and (**d**) EA (e = 54%).

To quantitatively assess the different strain localizations and damage behaviors of the two alloys, the distributions of the dissipated plastic energy along two selected paths on the front plane of the RVE models for the two alloys, indicated by arrowed dashed lines in the corresponding IPF maps in Figure 13a and Figure 14a, were plotted and are shown in Figure 15. A distinct localization of the dissipated plastic energy was evident in the LA curves. Conversely, it appeared to be relatively more uniformly distributed in the case of the EA. To quantitatively evaluate the concentration of the dissipated plastic energy, the ratio of the highest value to the average value, denoted by $\xi =G_{p}^{max}/{\bar{G}}_{p}$, can be defined and utilized. The ξ values were 3.44 and 2.46 for the LA and EA, respectively, at an engineering strain of *e* = 10%, reflecting the severe localization of the damage behavior in the LA (Figure 15a,c). This severe damage localization can be attributed to the undesirable microstructural morphology, highlighting the importance of tailoring the microstructural features by optimizing the LPBF processing parameters to improve the ductility of the LA [3,4,10].

## 5. Conclusions

In this study, a microstructure-based crystal plasticity model incorporating a ductile fracture criterion was developed to investigate the micromechanical deformation and damage behaviors of conventionally extruded and additively manufactured SS316L stainless steels during tensile loading. The following conclusions can be drawn from our combined experimental and numerical investigations:

1. The EA exhibited an equiaxed grain morphology with a rotated cube and Goss texture, whereas the LPBFed alloy (LA) exhibited a partially elliptic morphology on the LD plane owing to the presence of the melt pool and an orthogonal path-like morphology on the ND plane from the scanning path. The grain size distribution of the LA is quite dispersed due to its hierarchical nature of the microstructure. During tensile deformation, the <110> texture along the LD in the EA favored twinning, whereas the <001> texture along the LD in the LA impeded twinning.
2. The LA exhibited a higher YS but lower UTS than the EA. The increased yield strength in the LA was attributed to the higher as-manufactured dislocation density and the solidification-enabled cellular subgrain cellular structures, resulting in reduced elongation. The presence of annealing twins and favorable texture in the EA contributed to its excellent elongation, along with a higher work-hardening rate than LA, owing to twin–dislocation interactions during plastic deformation.
3. A mapping algorithm was developed to create microstructure-based RVEs by considering the real microstructures of alloys in crystal plasticity simulations. The developed CPFE model could accurately reproduce the tensile stress–strain behaviors and the evolution of crystallographic textures in both alloys by using a small number of modeling parameters.
4. Microstructure-based crystal plasticity modeling results revealed that the stress concentration, strain localization, and damage accumulation behavior in the EA were generally uniform, whereas those in the LA were severely localized around sites with a high density of local initial dislocations and numerous HAGBs. The higher tendency for strain localization and damage accumulation led to a lower elongation observed in the LA compared to that of the EA.

## Figures and Tables

**Table 1:** Nominal chemical composition of SS316L powders (in wt.%).

C	Si	Mn	P	S	Cr	Ni	Mo	Fe
0.03	0.75	2.00	0.05	0.03	17.00	12.00	2.50	65.65

**Table 2:** A 0.2% yield strength (YS), ultimate tensile strength (UTS), and tensile elongation (fracture strain) of the two alloys.

	YS (MPa)	UTS (MPa)	Elongation (%)
EA	328	604	71
LA	516	612	44
[4]	590	700	68
[11]	648	745	52
[38]	412	511	18

**Table 3:** Calibrated hardening parameters for the {111}<110> slip system of the studied alloys.

	$h_{0}$ (MPa)	$\tau_{c}^{\alpha }$ (MPa)	$\tau_{s}$ (MPa)	a	*q*
EA	1300	167	356	2.5	1.4
LA	1300	186	420	2.5	1.4

## Data Availability

The raw data supporting the conclusions of this article will be made available by the authors on request.
